# The effect of insert conformity and material on total knee replacement wear

**DOI:** 10.1177/0954411913513251

**Published:** 2013-12-02

**Authors:** Abdellatif Abdelgaied, Claire L Brockett, Feng Liu, Louise M Jennings, Zhongmin Jin, John Fisher

**Affiliations:** 1 Institute of Medical and Biological Engineering, School of Mechanical Engineering, University of Leeds, Leeds, UK; 2 Institute of Advanced Manufacturing Technology, School of Mechanical Engineering, Xi’an Jiaotong University, Xi’an, P.R. China

**Keywords:** Total knee replacements, insert conformity, wear, conventional ultra-high molecular weight polyethylene, moderately cross-linked ultra-high molecular weight polyethylene

## Abstract

The mean average life is increasing; therefore, there is a need to increase the lifetime of the prostheses. To fulfil this requirement, new prosthetic designs and materials are being introduced. Two of the design parameters that may affect wear of total knee replacements, and hence the expected lifetime, are the insert conformity and material. Computational models have been used extensively for wear prediction and optimisation of artificial knee designs. The objective of the present study was to use a previously validated non-dimensional wear coefficient-based computational wear model to investigate the effect of insert conformity and material on the predicted wear in total knee replacements. Four different inserts (curved, lipped, partial flat and custom flat), with different conformity levels, were tested against the same femoral and under two different kinematic inputs (intermediate and high), with different levels of cross-shear. The insert bearing materials were either conventional or moderately cross-linked ultra-high molecular weight polyethylene (UHMWPE). Wear predictions were validated against the experimental data from Leeds knee simulation tests. The predicted wear rates for the curved insert (most conformed) were more than three times those for the flat insert (least conformed). In addition, the computationally predicted average volumetric wear rates for moderately cross-linked UHMWPE bearings were less than half of their corresponding conventional UHMWPE bearings. Moreover, the wear of the moderately cross-linked UHMWPE was shown to be less dependent on the degree of cross-shear, compared to conventional UHMWPE. These results along with supporting experimental studies provide insight into the design variables, which may reduce wear in knee replacements.

## Introduction

An increasing number of joint replacement operations are being carried out every year in the United Kingdom, over 160,000 joint replacement operations per year in England and Wales. In 2012, more than 85,000 knee joint replacement operations were carried out in England and Wales.^1^ During knee replacement operations, the damaged bearing surfaces are being replaced by artificial ones. The two main objectives of the replacement operation are eliminating pain and restoring the joint function.^2^

As the mean average life expectancy is increasing, more designs and materials are required with lower wear, to improve the performance and increase the expected lifetime of the replacements. One of the design parameters that may affect wear of total knee replacements (TKR) is the insert conformity.^3,4^ The conformity of the bearing surfaces has been one of the main focuses of research in artificial knee joint designs since the publication by Bartel et al.^5^ More conforming designs have been favoured to reduce the contact stress and structural wear. More recent clinical, experimental and computational studies have again shown that insert conformity is an important parameter in total joint replacement wear. Less conforming designs may reduce surface wear, provided that the contact stress does not exceed the fatigue limit of the material, in which case fatigue wear mechanisms such as delamination may occur. However, the improvement in stability and mechanical properties of polyethylene materials has led to an increase in the fatigue limit.^4,6,7^

Cross-linking has been introduced to reduce wear of ultra-high molecular weight polyethylene (UHMWPE).^8^ Many studies, either in vitro or in vivo, have approved significant advantages for cross-linked UHMWPE over conventional UHMWPE.^9–11^ Cross-linking not only improves the wear resistance but also affects the oxidative stability and fatigue crack propagation.^12,13^ Different radiation doses and methods, fabrication techniques and heat treatments may result in different mechanical properties. Moderately, cross-linking improves the wear resistance and allows the mechanical properties to be maintained.^14,15^

Computational wear modelling has offered an alternative attractive approach to the experimental testing, allowing substantially reduced cost and time.^16^ Utilising the simplified Archard’s wear law,^17^ based on the sliding distance and load, computational wear models have been developed to predict material loss caused by wear.^18,19^ The contact area and contact pressure, rather than load, were used in a modified version of Archard’s wear law to optimise the TKR designs.^20^ More recently, based on the idea that wear volume is proportional to the contact area and sliding distance, a new wear formula has been applied to computational wear models.^4,6,21,22^ The computational wear predictions from the new wear formula have been validated against the experimental measurements, and the results were in good agreement.^21,22^

The objective of the present study was to use a previously described and validated non-dimensional wear coefficient–based computational wear model^21^ to investigate the effect of insert conformity and material on the predicted wear in total fixed human knee replacements.

## Materials and methods

**Figure 1.**
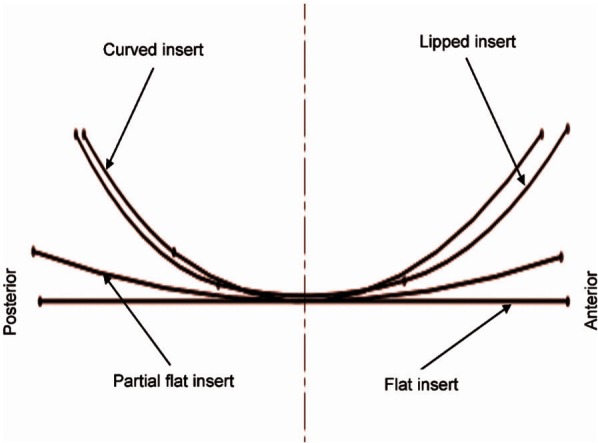
Schematic diagram for different insert types with different conformities.

The effect of insert conformity on the predicted wear in TKRs was investigated. Four different insert designs, with different conformity levels (Figure 1), were tested. The DePuy TKR Sigma fixed femoral component (DePuy International Ltd, UK) was run against custom flat, partial flat and lipped and curved inserts. The Sigma curved and lipped inserts were based on size 3 geometry with a thickness of 10 mm (DePuy International Ltd). The partial flat insert was based on the Sigma High Performance Partial Knee, while the custom flat insert was created by flattening the contact surface of the partial flat insert.

**Figure 2.**
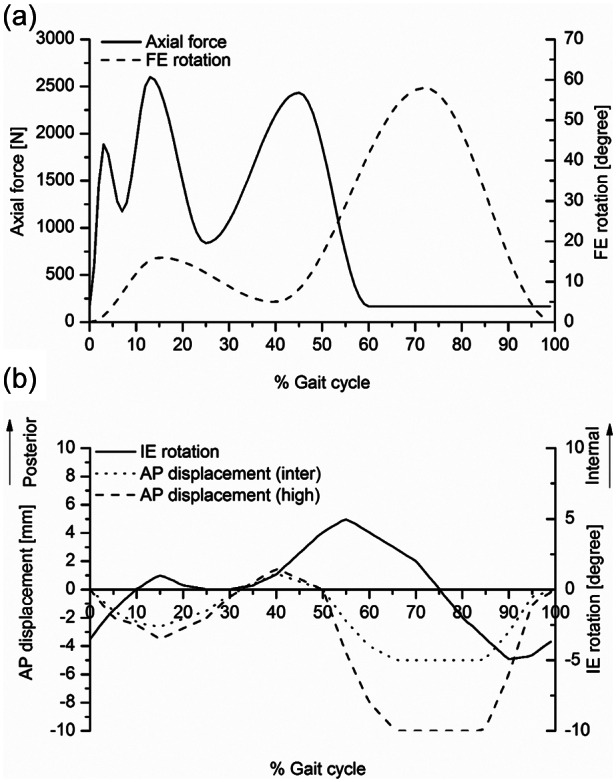
Knee simulator input profiles: axial force (N) and FE (°): (a) AP displacement (mm) and (b) IE rotation (°). FE: flexion–extension; AP: anterior–posterior; IE: internal–external.

Two different kinematic inputs, intermediate kinematic and high kinematic inputs, were used.^23^ The difference between intermediate and high kinematics was the level of anterior–posterior (AP) translation, being the maximum of 5 mm in the intermediate condition and 10 mm in the high kinematic condition, as shown in Figure 2. The AP displacement and internal–external (IE) rotation in the high kinematics were based on the kinematics of the natural knee, while the AP displacement in the intermediate kinematics was approximately half the magnitude of the high displacement. The axial load and flexion–extension (FE) rotation were based on the ISO standard kinematics for TKRs.^23^

Each design and each kinematic input were run for 3 million cycles in the computational model. The computational framework used in the present study was validated against the experimental results, for the conventional UHMWPE curved and flat inserts, elsewhere.^21^ The computational wear model for the knee implants was based on the contact area (*A*), sliding distance (*S*) and an independent experimentally determined non-dimensional wear coefficient (*C*) to calculate the volume wear loss (*W*) as

(1) W=C×A×S

The experimentally determined non-dimensional wear coefficient is a function of cross-shear ratio (*CS*), but not the applied nominal contact pressure.^21,22^ In multidirectional motion, the increased wear resistance in the main motion direction, due to strain hardening of the articulating polyethylene surface in that direction, is accompanied by depreciation in wear resistance in the direction perpendicular to that direction.^24^ In order to account for the contrary effect of strain hardening in polyethylene, the cross-shear ratio was defined based on the unified theory of wear and frictional work by Wang^24^ and the work by Kang et al.,^25^ as the frictional work component perpendicular to the principal molecular orientation (PMO) direction (*E_cross-shear_*), divided by the total frictional work (*E_total_*), thus

(2) $$\mathrm{CS}=\frac{E_{\mathrm{cross}-\mathrm{shear}}}{E_{\mathrm{total}}}$$

The relations between the non-dimensional wear coefficient and cross-shear ratio were determined from independent experimental pin-on-plate wear studies by Abdelgaied et al.^21^, Kang et al.^25^ and Abdelgaied et al.,^26^ and are given in equations (3) and (4), for conventional and moderately cross-linked UHMWPE materials, respectively. These independent experimental pin-on-plate studies determined the material wear coefficient as an input to the model

(3) $$C=10^{-8.895}{\left(8.517\times 10^{-5}+9.365\times \mathrm{CS}\right)}^{0.148}$$

(4) $$C=10^{-8.964}{\left(3.482\times 10^{-6}+2.057\times \mathrm{CS}\right)}^{0.191}$$

Compressive creep model, with appropriate parameters, for the conventional UHMWPE was derived from the curve-fitted experimental data reported by Lee and Pienkowski,^27^ as shown in equation (5). The calculated creep strains were modelled as inelastic, and the amount of creep recovery was assumed to be 0.5^28^

(5) $$\delta_{\mathrm{creep}}=\left[3.491\times 10^{-3}+7.966\times 10^{-4}\left(\log(t)-4\right)\right]\sigma_{\mathrm{av}}h$$

where *t* is time (min), *σ_av_* is the average pressure (MPa) and *h* is the thickness (mm).

Although it was reported that cross-linking may lead to a depreciation in creep performance,^29^ this reduction in creep properties can be alleviated by improving the cross-linking procedure,^30^ or using lower degrees of cross-linking.^31^ However, the exact creep properties of moderately cross-linked UHMWPE have not been reported in the literature. Moreover, it was reported that creep had a little influence on the computationally predicted volumetric wear rates.^21,32^ So that, the effect of creep in moderately cross-linked UHMWPE was assumed to be low and neglected, rather than assuming arbitrary creep values.

The conventional UHMWPE was modelled as isotropic elastic–plastic material in ABAQUS (ABAQUS 6.9-EF1), using the DePuy true stress–strain data (DePuy International Ltd), reported by Godest et al.,^33^ with a modulus of elasticity of 463 MPa and a Poisson’s ratio of 0.46.^34^ The moderately cross-linked UHMWPE was modelled using the stress–strain data with a modulus of elasticity of 673 MPa and a Poisson’s ratio of 0.46 (DePuy International Ltd). The mesh sensitivity study resulted in total number of elements of 18,314; 21,233; 40,537 and 42,777, for the flat, partial flat, lipped and curved inserts, respectively, using modified quadratic tetrahedral elements (C3D10M). The femoral component was modelled as a rigid body.^35^ The isotropic penalty contact was used to define the surface-to-surface contact between the tibial and femoral contact surfaces with a coefficient of friction of 0.04.^33^ The polyethylene insert contact surface was updated every 100,000 cycles for the first 500,000 cycles and then every 500,000 cycles, to account for the large creep in the early stages.^32^ The predictions for the wear rates from the computational model were compared to independently generated and published experimental wear rates from full knee joint simulator studies.

## Results

**Figure 3.**
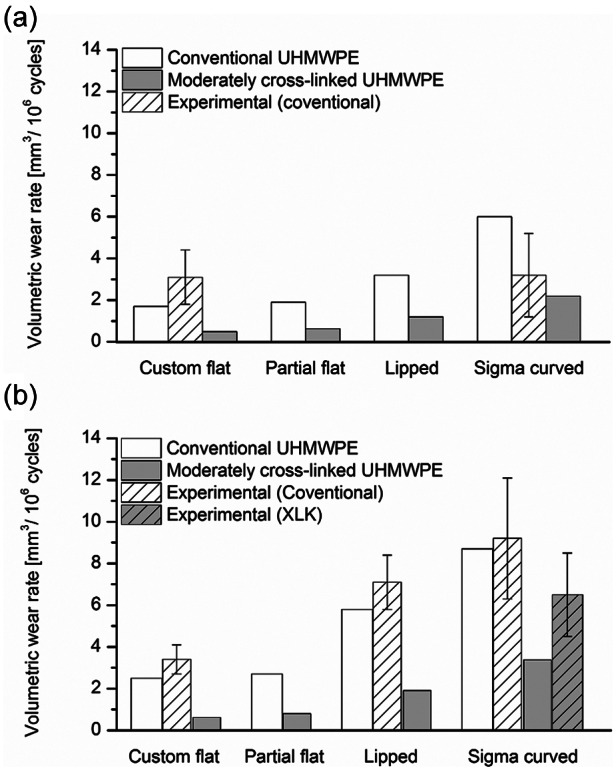
Experimental^4,7,36,37^ (mean ± 95% confidence interval) and computational volumetric wear rates (mm^3^/million cycles) for different fixed bearing inserts, with conventional and moderately cross-linked UHMWPE materials, under (a) intermediate and (b) high kinematic inputs. UHMWPE: ultra-high molecular weight polyethylene; XLK: cross-linked ultra-high molecular weight polyethylene

The predicted computational wear rates for different inserts, with conventional and moderately cross-linked UHMWPE bearing materials, under intermediate and high kinematic inputs, are shown in Figure 3. The conventional UHMWPE computational average wear rates for the flat, partial flat, lipped and curved inserts were 1.7, 1.9, 3.2 and 6 mm^3^/million cycles (under intermediate kinematic inputs) and 2.5, 2.7, 5.8 and 8.7 mm^3^/million (under high kinematic inputs), respectively. The corresponding predicted values for the moderately cross-linked UHMWPE inserts were 0.5, 0.63, 1.2 and 2.2 mm^3^/million cycles (under intermediate kinematic inputs), and 0.61, 0.8, 1.9 and 3.4 mm^3^/million (under high kinematic inputs), respectively.

The computationally predicted wear rates, for different inserts and different bearing materials, are compared to the available experimental results from the University of Leeds’ knee simulator for validation in Figure 3. The experimental wear rates for the Sigma curved insert with conventional UHMWPE were 3.2 ± 2 and 9.2 ± 2.9 mm^3^/million cycles under intermediate and high kinematic inputs, respectively.^36^ The measured experimental wear rate for the moderately cross-linked UHMWPE curved insert, under high kinematic inputs, was 6.5 ± 2 mm^3^/million cycles.^37^ The experimentally measured wear rate for the lipped insert with conventional UHMWPE material, under high kinematic inputs, was 7.1 ± 1.3 mm^3^/million cycles.^7^ The conventional UHMWPE flat insert had experimental wear rates of 3.1 ± 1.3 and 3.4 ± 0.7 mm^3^/million cycles, under intermediate and high kinematic inputs, respectively.^4^

**Figure 4.**
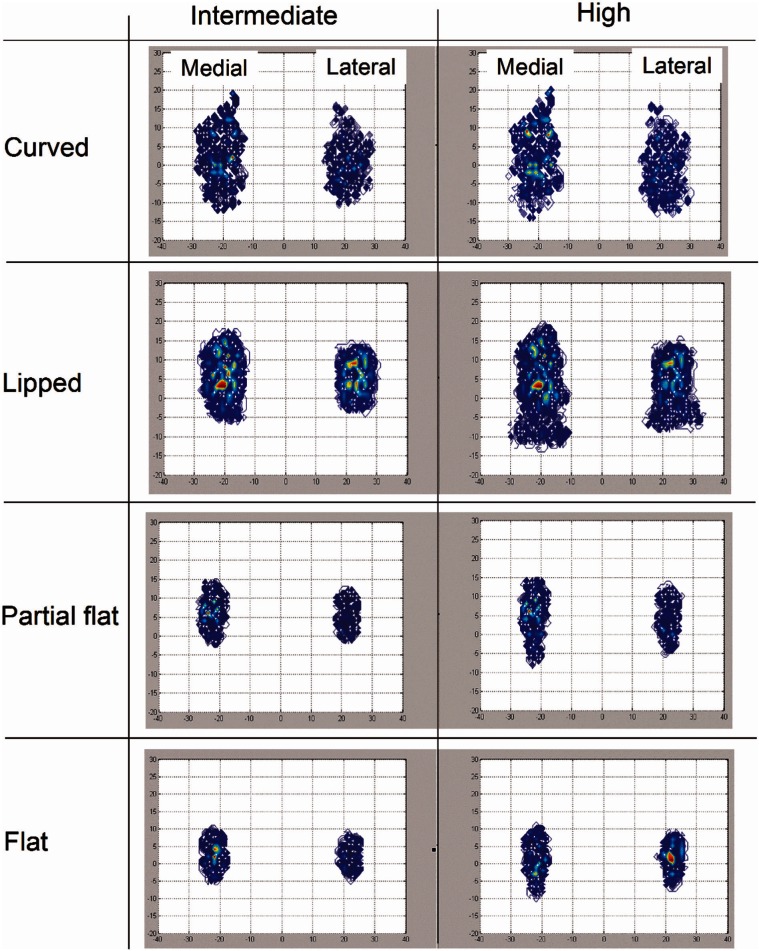
Computationally predicted wear scars for different fixed bearing inserts, with conventional UHMWPE bearing material, under intermediate and high kinematic inputs.

The computationally predicted wear scars (wear patterns) for different inserts, with conventional UHMWPE bearing material, under intermediate and high kinematic inputs are shown in Figure 4. The corresponding predicted wear scars for the moderately cross-linked UHMWPE inserts were similar in shape to those shown for the conventional UHMWPE inserts and are not shown in this article. For the same insert type and under the same kinematic input, the medial condyle wear pattern was shown to be larger than the lateral condyle one.

**Figure 5.**
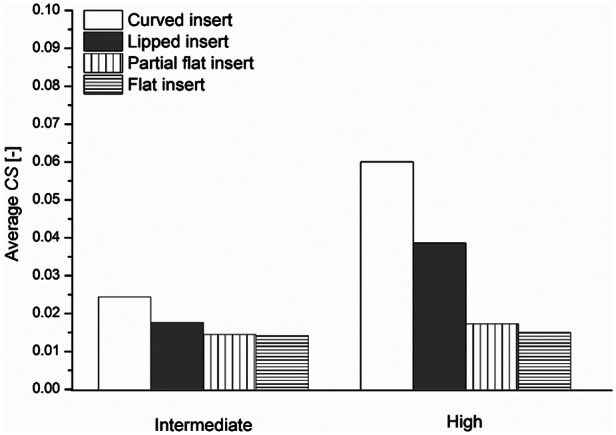
Effect of insert conformity on the predicted average *CS*, for the conventional UHMWPE inserts under intermediate and high kinematic inputs.

The computationally predicted average *CS*s for different inserts, with conventional UHMWPE bearing material, under intermediate and high kinematic inputs are shown in Figure 5. Decreasing the conformity level, by changing the bearing insert from curved insert (high conformity level) to flat insert (the lowest conformity level), decreased the predicted average *CS* under intermediate and high kinematic inputs. The predicted average *CS*s for the Sigma curved and flat inserts were 0.024 and 0.014 under intermediate kinematic inputs and 0.06 and 0.015 under high kinematic inputs, respectively.

## Discussion

The effects of insert conformity and material on the predicted average computational wear rates for the curved, lipped, partial flat and custom flat inserts were investigated, when articulating against the Sigma curved femoral component. The tests were run under intermediate and high kinematic inputs, up to 3 million cycles for each condition. The results showed that under both intermediate and high kinematics, the less conforming geometries had the lower predicted wear. This reduction in wear, as the conformity and contact area are reduced, is shown experimentally on the full joint simulator and predicted by the model applies for surface wear mechanisms, up to the point where the fatigue limit of the materials and additional fatigue wear mechanisms are initiated. This effect is contrary to some historical studies of knee prostheses, where with oxidised and degraded polyethylene, the fatigue limit was exceeded and the reduction in conformity and contact area and resulting increase in contact stress resulted in initiation of fatigue wear mechanisms such as delamination and an increase in fatigue wear.^38,39^

The former trend can be explained by the change in *CS* and contact area. The wear patterns for different inserts, summarised in Figure 4, showed that the higher the conformity, the higher the contact area between the insert and the femoral bearing. The dimensionless wear coefficient–based computational wear model is contact area and *CS* dependent (equation (1)). The directly proportional relationship between the volumetric wear and contact area in the current model means that under controlled kinematic inputs, the lower the contact area, the lower the wear. In addition, under the same kinematic inputs, the higher the conformity, the higher the *CS*, as shown in Figure 5, and hence the higher the wear. Increasing the conformity between the femoral and the insert increases the contact radius, with higher IE motion at the outer nodes, and results in higher *CS*s. The predicted average wear rates for the curved insert (high conformity level) were more than three times the custom flat insert ones (the lowest conformity level), under both intermediate and high kinematic inputs and different bearing materials. It is recognised that in the knee joint in the body, the actual kinematics are controlled by biomechanical forces, soft tissue constraints and insert conformity. So that, the same kinematic inputs assumption for different inserts with different levels of conformity might not be reflective of the knee joint. However, lower conforming knee joint geometries are being used in partial/unicompartmental knee replacements where natural joint biomechanics and soft tissues are retained, which control the knee and prevent excess motion.

The direct proportional relationship between the conformity level and the contact area, found in the present study, is consistent with the study by Bartel et al.^5,34^ The higher contact areas accompanying the higher conformity levels in the present study is another way to express the inversely preoperational relationship between the contact stress and the conformity level (under the same applied load).^5,34^ Although the importance of the conformity in the knee bearing designs has been recognised, different conclusions have been made. The rationale from Bartel et al.^5,34^ was to reduce the contact stress and limit the structural wear by increasing the conformity,^5^ whereas our study suggests otherwise: to reduce the conformity level to a certain extent, while keeping the contact stress within the strength limit. As a result of the improved material properties and the recognition of the importance of wear, as well as wear as a function of contact area, wear can be reduced using less conforming bearing surfaces because of the reduction in the contact area. Therefore, it is possible to choose an optimal conformity level to achieve a balance between the fatigue strength limit and wear requirements.

Contrasting the predicted average wear rates under different kinematics, the predicted average wear rates under high kinematics were approximately 1.5 times the corresponding predicted average wear rate under intermediate kinematics, for the same insert. The high kinematic inputs had larger wear patterns (contact areas) than the intermediate kinematics, for the same insert (Figure 4). In addition, changing the kinematic inputs from intermediate to high, for the same insert, increased the average *CS* (Figure 5) and accordingly the predicted wear.

**Figure 6.**
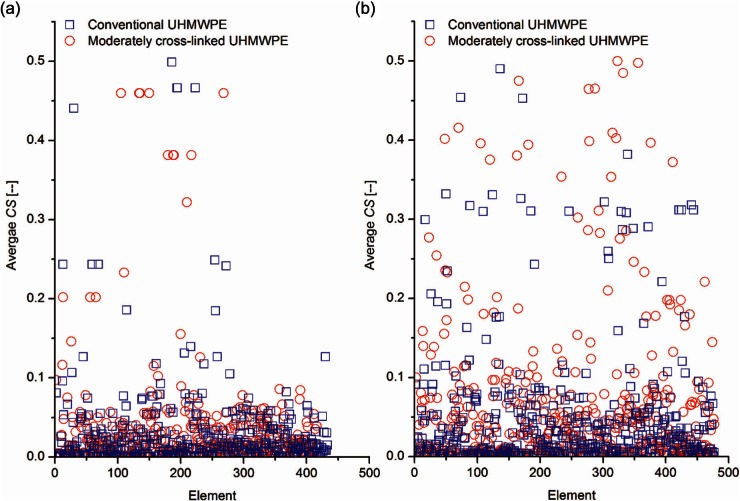
Effect of insert bearing material on the predicted average elemental *CS* for the Sigma curved insert under (a) intermediate and (b) high kinematic inputs. UHMWPE: ultra-high molecular weight polyethylene.

The computationally predicted average volumetric wear rates for moderately cross-linked UHMWPE bearings were lower than half their corresponding conventional UHMWPE bearings, under both intermediate and high kinematic inputs, as shown in Figure 3. This finding is consistent with the in vivo measurements.^10,40^ The reduction in volumetric wear rate with the change in bearing material was attributed to the associated changes in the experimental wear parameters, contact area and *CS*. The moderately cross-linked UHMWPE experimentally measured wear parameters, which were inputs to the computational models, were lower than those measured for conventional UHMWPE material. In addition, changing the insert bearing material from soft (conventional UHMWPE) to hard (moderately cross-linked UHMWPE) materials reduced the contact area during articulation. Moreover, different levels of deformations associated with different bearing materials resulted in different radii of contact between the femoral and the insert contact surfaces, and hence different *CS*s, as shown in Figure 6.

On the wear scar, the conventional and moderately cross-linked UHMWPE materials’ wear scars, for the same insert and under the same kinematic inputs, were similar. This wear pattern similarity contributes to the similar wear track, under the same kinematic inputs, not the wear degree. The difference in wear pattern between the medial and lateral condyles was mainly attributed to the loading axis offset in the medial direction, and to the difference in curvature between the medial and lateral condyles, in both the insert and the femoral contact surfaces.^4^

Contrasting the effect of kinematic inputs on the predicted average wear rates, the effect of kinematic inputs on the predicted average wear rates in moderately cross-linked UHMWPE bearings was lower than that for conventional UHMWPE, which shows less dependence of the moderately cross-linked UHMWPE wear on the degree of cross-shear, compared to the conventional UHMWPE.^11,41^

The computationally predicted volumetric wear rates were compared to the corresponding experimental wear results^4,36,37^ in Figure 3. The experimental results showed similar effects for insert conformity and bearing material on wear to that was shown computationally. The differences in wear rates between the computational and experimental results may be attributed to the experimental inputs (wear coefficients) to the model. The pin-on-plate wear studies conducted to calculate the experimental wear coefficients were run under constant load and against very smooth counterfaces. On the contrary, the experimental simulators were run under dynamic loading conditions and against cast cobalt chrome femoral surfaces. Additionally, two different sets of experimental measurements were used for validation. However, two sets of experimental data from the knee simulator studies that are 4 years apart showed a significant difference in the predicted wear.^37^ Most importantly, the computational model was able to describe even the small changes in wear rates with the change in insert conformity, kinematic inputs or bearing material. The computational wear model described as small changes as 0.11 mm^3^/million cycles (~18% change) with the change in kinematic inputs, from high to intermediate, for the flat insert with moderately cross-linked UHMWPE material. Nonetheless, the model not only described the difference in wear rate between the extreme levels of conformity (flat and curved inserts), but also between intermediate levels of conformity (partial flat and lipped inserts). The applicability of the computational model to four different insert conformities with two different bearing materials and under two different kinematic inputs emphasises the ability of the model to describe the change in wear with the change in kinematics, design (in terms of the geometry of the tibial insert, that is, different insert conformities) and material and to provide a more robust modelling platform.

Although the conventional UHMWPE computational model showed that up to 90% of the early-stage linear deformations were attributed to creep, creep deformation of moderately cross-linked UHMWPE was assumed to be minimal and was neglected, due to the lack of creep data reported for the cross-linked UHMWPE material. However, the contribution of creep to the volumetric wear of conventional UHMWPE was less than 5%. Moreover, our results are only valid for the tested contact pressure range from the current inserts and femoral combinations and bearing materials, and under the specified loading conditions (the maximum predicted average elemental contact pressure ranged from 30 to 40 MPa and from 45 to 60 MPa, according to the insert type, for conventional and moderately cross-linked UHMWPE bearing materials, respectively). For these specified conditions, the surface wear mechanism is dominated. Under higher contact pressures, different wear mechanisms might take place.

## Conclusion

The results showed that a potential method for increasing the expected TKR lifetime might be to introduce less conforming knee replacements. In addition, introducing moderately cross-linked UHMWPE as a bearing material resulted in considerable reduction in the predicted wear, up to 50%, for all insert types. Moreover, the moderately cross-linked UHMWPE wear was shown to be less dependent on the degree of cross-shearing, compared to the conventional UHMWPE material.
